# Tailoring Cognitive Behavioral Therapy to Subtypes of Voice-Hearing

**DOI:** 10.3389/fpsyg.2015.01933

**Published:** 2015-12-21

**Authors:** David Smailes, Ben Alderson-Day, Charles Fernyhough, Simon McCarthy-Jones, Guy Dodgson

**Affiliations:** ^1^Department of Psychology, Durham UniversityDurham, UK; ^2^Department of Psychology, Leeds Trinity UniversityLeeds, UK; ^3^Department of Psychiatry, Trinity College DublinDublin, Ireland; ^4^Early Intervention in Psychosis, Northumberland, Tyne and Wear NHS Foundation TrustAshington, UK

**Keywords:** hallucinations, voice-hearing, psychosis, schizophrenia, cognitive behavioral therapy

## Abstract

Cognitive behavioral therapy (CBT) for voice-hearing (i.e., auditory verbal hallucinations; AVH) has, at best, small to moderate effects. One possible reason for this limited efficacy is that current CBT approaches tend to conceptualize voice-hearing as a homogenous experience in terms of the cognitive processes involved in AVH. However, the highly heterogeneous nature of voice-hearing suggests that many different cognitive processes may be involved in the etiology of AVH. These heterogeneous voice-hearing experiences do, however, appear to cluster into a set of subtypes, opening up the possibility of tailoring treatment to the subtype of AVH that a voice-hearer reports. In this paper, we (a) outline our rationale for tailoring CBT to subtypes of voice-hearing, (b) describe CBT for three putative subtypes of AVH (inner speech-based AVH, memory-based AVH, and hypervigilance AVH), and (c) discuss potential limitations and problems with such an approach. We conclude by arguing that tailoring CBT to subtypes of voice-hearing could prove to be a valuable therapeutic development, which may be especially effective when used in early intervention in psychosis services.

## Introduction

Auditory verbal hallucinations (AVH), or “voice-hearing,” refers to the experience of hearing a voice in the absence of an appropriate external stimulus and which “has a sufficient sense of reality to resemble a veridical perception, over which the subject does not feel s/he has direct and voluntary control, and which occurs in the awake state” (David, 2004, p. 110). Whilst some voice-hearers report that their experiences have a broadly positive impact on their lives (e.g., Jenner et al., 2008) and a significant proportion do not have a need for psychiatric or psychological help (Johns et al., 2014), others are very distressed and impaired by their experiences. When such people come into contact with psychiatric or psychological services, they are typically diagnosed with a psychotic disorder, such as schizophrenia (although AVH are reported by people with other mental health problems, e.g., mood disorders, borderline personality disorder, and dissociative disorders; Larøi et al., 2012), and they receive medication, psychological therapy, or a combination of both, to help them cope with their voice-hearing and any co-morbid experiences (e.g., persecutory delusions).

Cognitive behavioral therapy (CBTp; e.g., Morrison et al., 2004) is the most well-known and widely researched psychological intervention for psychosis (McCarthy-Jones et al., 2015). CBTp typically aims to reduce the distress associated with psychotic experiences, rather than attempting to reduce the frequency of those experiences (Morrison and Barratt, 2010). To achieve this aim, a wide range of different techniques are used and, thus, CBTp refers to a relatively broad range of psychological interventions (Thomas et al., 2014; Thomas, 2015). As a result, there is considerable debate about what the essential components of CBTp are (Morrison and Barratt, 2010) and about which interventions “count” as CBTp (e.g., Lincoln, 2010; McKenna et al., 2010; Laws et al., 2014). For the purposes of this paper, we consider CBTp to refer to the interventions outlined in treatment manuals such as Morrison et al. (2004), Kingdon and Turkington (2005), and Beck et al. (2009), which focus on alleviating the distress associated with AVH through changing a voice-hearer's appraisal of their AVH, based on a developmental, individualized formulation. This aim is achieved through the normalization of psychotic experiences, the use of behavioral experiments to test unhelpful beliefs about voices, the development of better coping strategies, the adoption of more effective emotion regulation strategies in place of unhelpful strategies (such as safety behaviors), and the revision of negative beliefs about the self. While these manuals were published between 6 and 11 years ago, they continue to guide treatment in many CBTp studies (e.g., Morrison et al., 2014).

CBTp is recommended by the National Institute for Health and Care Excellence (NICE) in the UK (National Institute for Health and Care Excellence (NICE), 2009, 2015), as well by bodies in the United States of America (Dixon et al., 2010) and Australia (Royal Australian and New Zealand College of Psychiatrists, 2005). In the UK, the recommendation that CBTp be offered to people who are diagnosed with a psychotic disorder is largely based on a series of meta-analyses that reported CBTp to be moderately effective in reducing the severity of the positive psychotic symptoms (e.g., Zimmerman et al., 2005; Wykes et al., 2008). However, these meta-analyses did not examine the effects of CBT on voice-hearing specifically (i.e., reductions in positive symptoms might refer to reductions in delusions, thought disorder, and/or hallucinations in any modality). Recent meta-analyses have found that that CBTp is effective at reducing the severity of auditory hallucinations (van der Gaag et al., 2014, Hedges' *g* = 0.49; Jauhar et al., 2014, Hedges' *g* = 0.34).

Despite these studies, there is good evidence to suggest that these effect sizes are not representative of CBT's true effect on voice-hearing. First, there is evidence that the effects of CBTp on positive symptoms are substantially reduced when potential sources of bias (e.g., failure to blind assessors to the treatment assignment of participants, researcher allegiance) in trials are taken into account (Jauhar et al., 2014). For example, Jauhar et al. reported that masking moderated the effect of CBT on positive symptoms (although see McKenna and Kingdon, 2014, for a critique of the way in which Jauhar et al. examined masking). In studies where masking appears to have been compromised (i.e., raters may not have been blind to the treatment allocation), there was a moderate, significant effect of CBTp on positive symptoms (Hedges' *g* = 0.57), while in studies where it appeared unlikely that masking was compromised, there was a non-significant effect of CBTp on positive symptoms (Hedges' *g* = 0.08). In terms of hallucinations, Jauhar et al. reported a large difference between the effect size of CBTp for auditory hallucinations between masked (Hedges' *g* = 0.18) and non-masked studies (Hedges' *g* = 0.91). Second, van der Gaag et al.'s meta-analysis has been criticized by Laws et al. (2014) for including studies which found large effect sizes for CBT, but (a) were small-scale pilot studies (e.g., McLeod et al., 2007), which some argue should be excluded from meta-analyses (Kraemer et al., 1998; Coyne et al., 2010), or (b) used a form of therapy (Avatar therapy; Leff et al., 2013) that some researchers and clinicians do not regard as a CBT intervention (Laws et al., 2014; Leff et al., 2014). It should be noted, however, that the concerns raised by Laws et al. are not shared by other researchers (e.g., some researchers argue that small studies should be included in meta-analyses, so long as they are methodologically sound in other ways, such as having adequate randomization and blinding procedures in place; Sackett and Cook, 1993; Schulz and Grimes, 2005). In summary, CBT has been shown to have small-to-moderate effects on voice-hearing, but these effects may be inflated because of methodological problems in some RCTs.

## Why isn't CBT more effective for voice-hearing?

One reason why CBT for voice-hearing may have limited effectiveness is that current interventions typically treat voice-hearing as a relatively homogenous experience in terms of underlying cognitive processes. While most researchers and clinicians acknowledge heterogeneity in voice-hearing and suggest that this must be addressed by interventions (e.g., they report that the interventions provided in a study were individually tailored treatments based on case formulations), it is unclear what specific facets of the diverse voice-hearing experience determine what kind of intervention is delivered. For example, the coping strategies and behavioral experiments employed in CBT for voice-hearing often refer to ways that a voice-hearer can interrupt inner speech, based on the assumption that if one is able to interfere with the production of inner speech, one can prevent the generation of the “raw material” of an AVH. This is demonstrated by one recent CBT for voice-hearing intervention, which included participants learning coping skills such as humming, singing, reading, reading out loud, and talking to someone (Zanello et al., 2014). Similar strategies that involve subvocalization are endorsed by Kingdon and Turkington (2005) and by Beck et al. (2009) as ways in which a voice-hearer may attempt to interrupt AVH. Meanwhile, Morrison et al. (2004) suggested that, to encourage a voice-hearer to consider the possibility that their voices may be internally generated, they should “conduct behavioral experiments using subvocalization” (p. 134) and observe how this interrupts their AVH. These approaches seem to imply that voices have their basis in a form of inner speech, which may be the case for some voice-hearers but not for others (Jones, 2010; McCarthy-Jones, 2012). Heterogeneity in the involvement of processes related to inner speech in voice-hearing may account for the variability in the success of this coping strategy. For example, subvocal counting has also been found to be an effective long-term intervention in less than a fifth of voice-hearers (Nelson et al., 1991).

Thus, it is possible that current CBT interventions for voice-hearing fail to address the range of different cognitive processes that underlie AVH. This could be considered to be the only reasonable strategy available to clinicians, given the enormous heterogeneity (e.g., Nayani and David, 1996; McCarthy-Jones et al., 2014b; Woods et al., 2015) of AVH reported by voice-hearers. However, analysis of the phenomenology of voice-hearing suggests that, from this huge diversity, it is possible to identify a meaningful set of subtypes of voice-hearing, for which one might be able to develop specific sets of treatments. In the next section we briefly review evidence supporting the existence of subtypes of AVH.

## Evidence for subtypes of voice-hearing

Despite the heterogeneity of AVH (e.g., Nayani and David, 1996; McCarthy-Jones et al., 2014b; Woods et al., 2015), the phenomenology of AVH reported by voice-hearers suggests that they can be divided into a relatively small number of subtypes. For example, Stephane et al. (2003) performed a cluster analysis of 21 phenomenological properties of AVHs reported by 30 participants (most of whom were diagnosed with schizophrenia), which indicated the existence of two subtypes. One subtype was characterized by repetitive, simple content (e.g., AVH consisted of repeatedly hearing one or two words), by clear acoustics, by hearing the voice in external space, by being accompanied by other hallucinations, and by recognition of the self as the source of the AVH. The other subtype was characterized by non-repetitive content, which was moderately to highly complex (e.g., AVH ranged from sentences to conversations), by an inner space location, by multiple voices, by a lack of clear triggers, and by a belief that the source of the AVH was another person. More recently, McCarthy-Jones et al. (2014b) performed a cluster analysis of 13 phenomenological properties of auditory hallucinations reported by 199 participants (most of whom, again, were diagnosed with schizophrenia), which suggested the existence of three subtypes of AVH (as well as a nonverbal auditory hallucinations subtype). The first AVH subtype, termed “Constant Commenting and Commanding AVH,” was characterized by repetitive commands, or almost constant commentary, and were typically in the first or third person. The second AVH subtype, termed “Own Thought AVH,” was characterized by content that was not directed at a person and was in the first person, by being similar to memory, and by possibly being one's own “voice” or thoughts. The third AVH subtype, termed “Replay AVH,” was characterized by being “identical to a memory of heard speech” (p. 229). While these two studies do not wholly concur on which subtypes of AVH may exist, they both indicate that it is possible to categorize AVH into a small number of subtypes.

Based, in part, on these findings, McCarthy-Jones et al. (2014a) tentatively suggested the existence of five subtypes of voice-hearing. The first, hypervigilance AVH occur when a person perceives the presence of a threat-related word or phrase in environmental noise (e.g., a young man may hear the insult “nonce” in the chatter of a crowd; Dodgson and Gordon, 2009). The second, memory-based AVH occur when processes normally involved in retrieving memories generate an intrusive verbal cognition (e.g., which resembles something derogatory said by a critical caregiver, or something said during a traumatic experience) and a person misattributes this to an external, non-self source. The third, inner speech-based AVH occur when processes normally involved in producing inner speech generate a cognition which a person misattributes to an external, non-self source. The fourth, epileptic AVH occur—by definition—in people with a diagnosis of epilepsy, appear to be a result of specific lesions in posterior temporal language areas, and differ in a number of important ways from the AVH reported by voice-hearers who do not have epilepsy (Serino et al., 2014). The fifth, deafferentation AVH occur when deafferentation-like changes occur in auditory cortex or other language processing regions, brought on by hearing loss (Cole et al., 2002), or social isolation (Hoffman, 2007). These changes are thought to elicit neural activity that creates internal, self-generated cognitions that are very difficult to distinguish from external, non-self-generated events, and so these cognitions are experienced as AVH.

## Implications of the existence of subtypes of voice-hearing

If these putative AVH subtypes can be reliably identified in voice-hearers, there are important implications for therapeutic interventions. For example, Jones (2010) claimed that different subtypes of voice-hearing may be caused by different neurobiological and/or cognitive mechanisms. If one accepts this claim, it is tempting to argue that different therapeutic interventions will be required for different subtypes, given that each intervention will have to address a different set of neurobiological alterations (if it is a pharmacological intervention) or of cognitive problems or biases (if it is a psychological intervention). This argument has received support from a small number of studies.

For example, Stephane et al. (2001) reported two cases of service-users who experienced AVH that were fixed and repetitive. Anti-psychotic medication appeared to be ineffective in reducing the frequency of these AVH. Given the nature of the voices reported by the two service-users (i.e., in some ways they were similar to the intrusive thoughts experienced in OCD), both were prescribed fluvoxamine (an anti-obsessional agent). In both cases, fluvoxamine appeared to be effective in reducing the frequency of AVH. Thus, Stephane et al. suggest that the AVH experienced by these two service-users may belong to an obsessional subtype of AVH, which differ from other AVH in terms of their fixed, repetitive content. Moreover, they argued that these AVH may have a distinct neural substrate, which can be modified by anti-obsessional rather than anti-psychotic medication. To take an example from clinical psychology, Kingdon and Turkington (1998) postulated the existence of four subtypes of psychosis—obsessional psychosis, drug-related psychosis, anxiety psychosis, and sensitivity psychosis—and described how interventions for AVH needed to be modified according to each subtype. For example, they suggested that obsessional AVH tend to occur when a person experiences a thought that they deem to be unacceptable (e.g., they have an unpleasant thought and think, “I would never think that and so I cannot be the source of that thought”). Accordingly, therapy for this type of AVH involved psychoeducation about the nature of unwanted, intrusive thoughts. In contrast, Kingdon and Turkington suggested that sensitivity AVH occur in people who struggle to cope with relatively minor stressors (e.g., moving away to university) and so therapy involved training of social skills and novel coping strategies that would enable a voice-hearer to better cope with minor stressors.

The idea of tailoring CBT to subtypes of AVH is not, therefore, entirely novel. However, none of the approaches that have encouraged clinicians to tailor therapy to subtypes of AVH have been formally evaluated. Moreover, these approaches have provided little theoretical basis for the interventions they propose and they have given little guidance on how a clinician should decide which intervention is right for whom. In the next section, we describe a novel CBT manual for voice-hearing, which contains clinician- and client-oriented information (a) showing how a subtype of AVH may be identified, (b) explaining how CBT should be modified according to the subtype of AVH that has been identified, and (c) providing a theoretical rationale for why CBT should be tailored in a particular manner for each subtype. The manual is concerned with three of the subtypes proposed by McCarthy-Jones et al. (2014a)—inner speech-based AVH, memory-based AVH, and hypervigilance AVH. The two other subtypes proposed by McCarthy-Jones et al.—epileptic AVH and deafferentation AVH—are not considered in the manual given that these experiences are probably much more amenable to biological than psychological interventions. In addition, McCarthy-Jones et al. propose that within the inner speech- and memory-based subtypes, further subtypes may exist (e.g., inner speech-based AVH could be divided into “own thought,” “novel,” and “obsessional”). However, in its present form, the manual does not consider these within-subtype distinctions (i.e., the manual would propose the same intervention for own thought inner speech-based AVH as for novel inner speech-based AVH).

## CBT for subtypes of voice-hearing

As in other forms of CBTp, the manual encourages the clinician to develop a shared problem list with a service-user and to respect their interpretation of their experiences (i.e., voices should not be dismissed as “just externally misattributed inner speech” or “just externally misattributed memories”). The manual (Dodgson et al., 2014; available on request) begins by trying to establish the subtype of AVH that a service-user is experiencing, primarily through questions about phenomenological properties of the voice (or voices) they hear. The clinician is encouraged to ask questions about the auditory properties of the voices (e.g., do they sound as if someone is speaking to you, or are they sometimes silent?), about whether the voice appears to originate from (e.g., inside or outside the head), about the length of the voice's utterances (i.e., short vs. long utterances), and about the identity of the voice. In addition to these questions about the phenomenology of the AVH, the service-user is asked a series of questions about the triggers of AVH and about the contexts in which AVH occur. For example, the voice-hearer is asked about where their attention is focused (e.g., internally, on their own thoughts and feelings, or externally, on other people) when they experience an AVH, about the situations they are in when they typically experience AVH (e.g., alone, in a quiet room or in a noisy room with lots of people), and about what emotions tend to precede the occurrence of an AVH.

The voice-hearer's answers to these questions should enable the clinician to come to a decision about the subtype of AVH the voice-hearer is experiencing. None of these questions are “diagnostic” of a person experiencing a particular subtype, but they can provide strong indications that a person is experiencing one subtype rather than another. For example, while both inner speech-based and memory-based AVH may sound as if they are sometimes coming from inside and sometimes from outside the head, hypervigilance AVH should only ever be experienced as coming from outside the head (Dodgson and Gordon, 2009; Garwood et al., 2015). Similarly, both memory-based and hypervigilance AVH are characterized by having repetitive content; the former because the AVH is based on a memory, which should stay relatively stable over time, the latter because this type of AVH is a product of a person scanning the environment for a particular phrase or set of phrases. However, if a voice-hearer reports that the content is similar to what was often said to them by, for example, an abusive parent, and that they tend to experience the voice when they are alone at home, this would suggest that they are experiencing memory-based AVH (given that hypervigilance AVH are typically experienced in noisy, social environments). Drawing on this information, the clinician should then develop an individualized longitudinal formulation with the voice-hearer, which explains how and why the AVH has developed, and which subtype of AVH the service-user is experiencing.

Based on the decision about what subtype of AVH a voice-hearer is experiencing, the clinician is encouraged to flexibly draw on a series of treatment options, which are based on current models of each subtype of AVH (e.g., Fernyhough, 2004; Waters et al., 2006; Dodgson and Gordon, 2009) or of related phenomena (e.g., intrusive memories in PTSD; Ehlers and Clark, 2000). While there is some overlap in the three treatment packages (e.g., affective problems are thought to play an important role in each subtype of AVH), there are important differences between each approach. The three treatment approaches are outlined below.

## CBT for inner speech-based AVH

Inner speech-based AVH are thought to occur when a person generates a cognition, using many of the process normally involved in generating inner speech, and misattributes that cognition to an external, non-self source (Frith and Done, 1988; Fernyhough, 2004). A number of cognitive mechanisms are hypothesized to play a role in the development of this type of AVH. First, a person is thought to generate a cognition that has a dialogic structure (i.e., it takes the form of a to and fro conversation, rather than a monolog), and that has the auditory qualities of another person's voice (Hoffman et al., 2008; for fuller accounts of the different forms inner speech can take and how this relates to voice-hearing, see Fernyhough, 2004; McCarthy-Jones and Fernyhough, 2011). Second, this cognition is thought to occur with little effort. Thus, it lacks one of the key characteristics (i.e., cognitive effort) that we use to identify self-generated cognitions from non-self-generated events (Johnson, 1997). Third, this cognition may have been subject to thought suppression, which can make the cognition feel even less self-generated and, ironically, increases the frequency of the intrusions (Salkovskis and Campbell, 1994). Fourth, some voice-hearers are thought to have a trait-like bias in their reality discrimination skills, so that they tend to misattribute internal, self-generated cognitions to an external, non-self source (Brookwell et al., 2013). Moreover, this bias can be exacerbated by negative affect (Hoskin et al., 2014; Smailes et al., 2014).

Psychoeducation for this subtype involves guided discovery in which voice-hearers are presented with information about (a) how inner speech develops, based on a Vygotskian (Vygotsky, 1934/1987) model; (b) the different forms of inner speech that we can experience; (c) how low effort cognitions are hard to identify as self-generated; (d) how ineffective thought suppression can be, and how it typically leads to a paradoxical increase in the suppressed thought; (e) how stress/negative affect can make it difficult to recognize cognitions as self-generated. Thus, a core aim of psychoeducation for inner speech-based AVH is to help a voice-hearer understand how diverse normal inner speech is and how, in some situations, we can feel that we have no control over the content of our thoughts.

The coping strategies suggested for inner speech-based AVH are, to some extent, similar to the coping strategies employed in traditional CBT for voice-hearing. For example, they involve activities that will block the phonological loop, such as soothing self-talk, humming, and singing to oneself. In addition, behavioral experiments that involve using inner speech to practice transforming either the content of the voice (into something positive) or the sound of the voice (from an unpleasant, dominant-sounding voice to a less powerful, even amusing voice) may help to reduce the distress experienced by voice-hearers as it shows ways in which they can try to control AVHs when they occur. Beyond activities focused on inner speech, the manual encourages the avoidance of thought suppression strategies and rumination, and the use of effective emotion regulation strategies, such as distraction and seeking social support.

### Case vignette

Philip had become acutely unwell, leading to a hospital admission, where he described a belief that he had been kidnapped by psychologists for an experiment. He believed that he had been placed in a false town that copied his home town and his parents and family were imposters. Philip could hear and see the psychologists, particularly at night. Treatment initially focused on reducing the risk to his parents and testing out his delusional beliefs, before the focus turned to his voices. Philip described being distressed by conversations with the psychologists and also intrusive critical voices. Philip's experiences were classified as inner speech-based AVH as they typically provided a running commentary on his activities and happened more often when he was alone and focused on his own thoughts. Therapy involved presenting an explanation of how inner speech develops and an exercise on various ways people can experience inner speech, including forms of inner speech that “sound” like other people's voices. The role of the phonological loop in a person's inner speech was described and Philip was encouraged to try to block the loop with humming and listening to music in his head. The success of these strategies increased his belief that the voices were similar to his inner speech. Philip was then encouraged to summon the psychologists in his mind and transform both their voice and appearance. He enjoyed forcing them into comic voices and appearances, which provided further evidence that they were similar to his inner speech and that he could exercise control over them. When Philip experienced the voice he became adept at blocking the phonological loop or transforming the voice into something comic, which reduced his distress and the voices started to reduce in frequency.

## CBT for memory-based AVH

Memory-based AVH are thought to occur when a person experiences an intrusive (typically unpleasant) verbal cognition, through many of the process normally involved in generating auditory memories, and misattributes it to an external, non-self source (McCarthy-Jones et al., 2014a). Again, a number of cognitive mechanisms are hypothesized to play a role in the development of this type of AVH, many of which are also involved in the development of inner speech-based AVH. First (and most obviously), a person is thought to experience an intrusive verbal cognition. The intrusive nature of the cognition may be a result of it being related to a memory that was encoded during a traumatic event. Memories of traumatic events are often encoded in a data-driven manner, rather than in conceptually-driven manner (Ehlers and Clark, 2000). That is, they are frequently encoded in terms of sensory impressions and perceptual characteristics, rather than in terms of context and meaning. As a result, these memories tend to be recalled involuntarily, as a result of perceptual or emotional cues rather than by intentional recall (Ehlers et al., 2004). Thus, by their very nature, these memories—or in this case, cognitions related to these memories—occur without any cognitive effort (i.e., they are triggered by being in a place that resembles the place where a traumatic event occurred), and so will be experienced as intrusive (hence participants who report high levels of data-driven processing at the time of a trauma are more likely to develop PTSD, or PTSD symptoms, than are participants who report low levels of data-driven processing at the time of a trauma; Murray et al., 2002; Halligan et al., 2003). Alternatively, the cognition may be related to a memory that was not encoded during a traumatic experience (e.g., it may be an unpleasant comment made repeatedly by a teacher at school), but given its negative content, it may have been subject to thought suppression. As described above, suppressed cognitions are more likely to rebound into consciousness, and so will be experienced as intrusive. Thus, through these mechanisms, a person will experience an intrusive cognition that lacks one of the key characteristics (i.e., cognitive effort) that we use to identify self-generated cognitions from non-self-generated events (Johnson, 1997). When these intrusions are experienced in the context of biased reality discrimination, they are experienced as AVH, rather than being identified as an internal, self-generated cognition.

Psychoeducation for this subtype involves guided discovery in which voice-hearers are presented with information about (a) how memory normally works; (b) how memories of traumatic experiences tend to differ from normal memories; (c) how low effort cognitions are hard to identify as self-generated; (d) how ineffective thought suppression can be, and how it typically leads to a paradoxical increase in the suppressed thought; (e) how stress/negative affect can make it difficult to recognize cognitions as self-generated. Thus, a core aim of CBT for memory-based AVH is to help a voice-hearer to understand that AVH can be seen as a relatively normal response to some type of traumatic experience.

Many of the coping strategies for memory-based AVH are drawn from interventions for PTSD (e.g., Ehlers et al., 2005), given that the memory intrusions experienced in PTSD and memory-based AVH can be considered similar phenomena (some would go so far as to say that these are sometimes the same phenomena; Read et al., 2005). The aim of these coping strategies is to reduce a person's reliance on the use of avoidant coping strategies (such as thought suppression, avoidance of reminders of a traumatic experience, and other safety behaviors), to encourage the use of effective emotion regulation strategies (e.g., distraction), and to change excessively negative appraisals and interpretations of a trauma and its consequences. Careful discussion of a traumatic event can help to achieve several of these aims (Smith et al., 2006). First, effective emotion regulation strategies can be employed when a service-user experiences high levels of distress during the discussion. Second, a service-user can learn that they are able to cope with the negative emotions that thinking about the trauma evokes. This is important as fear of not being able to cope with these emotions may have been one reason for adopting avoidant strategies. Third, this discussion, and the therapist's reactions during the discussion, can be a way in which a service-user can disconfirm some of their negative trauma-related beliefs (e.g., “It was my fault,” “I will never get over this experience,” “People will think bad things about me if they know about what happened”). In addition, it is possible that through this discussion, memories and other cognitions related to the traumatic event can begin to be re-integrated into everyday autobiographical memory meaning that trauma-related memories should be less likely to be unintentionally recalled as a result of sensory or emotional cues (Conway, 1997; Ehlers and Clark, 2000).

### Case vignette

Grant had survived sexual, physical, and emotional abuse in a children's home but had started to experience voices in his early adulthood. These were constant and highly distressing and disabling, even when on high levels of medication. Grant had been reluctant to engage with therapy, but agreed to attend when the therapist provided information about the prevalence of voice-hearing in people who had experienced multiple forms of abuse, suggesting that voice-hearing may be a problem linked to his abusive past. Reducing the effects of voice-hearing on his functioning was his initial goal for therapy. An initial assessment of his voice-hearing suggested that Grant experienced inner speech-based AVH, with intrusive thoughts that mirrored his beliefs about himself, which were worse when he was unoccupied. However, when questioned about his first experience of voice-hearing, he described hearing footsteps and laughter at the end of a corridor. Grant was already aware of the link between trauma and voice hearing and quickly made the link with his experiences of lying awake at night listening out to see if abusers would come to his room. When he understood that his first experience of voice-hearing had been similar to experiencing an intrusive memory, Grant was able to understand that his current experiences were also self-generated and that the content was thematically similar to the comments of his abusers. With this increased insight, he was able to engage in specific distraction techniques which increased his sense of control over his voices, reduced the distress associated with this voices, and is starting to experience his voices less often.

## CBT for hypervigilance AVH

Hypervigilance AVH are thought to occur when a person is concerned that others hold specific negative beliefs about them (e.g., that they are a pedophile). As a result, a person becomes very anxious, scans the environment for comments related to those beliefs, and begins to misinterpret environmental noise (e.g., traffic noise, crowd noise, or mechanical hums) as containing those comments (see Dodgson and Gordon, 2009). In part, these “false alarms” appear to occur because arousal shifts the balance of perceptual systems, so that top-down processes have a larger influence on our perceptions (Dudley et al., 2014).

Psychoeducation for this subtype involves guided discovery in which voice-hearers are presented with information about (a) the role of top-down influences on perception; (b) how our perceptual systems have evolved to help us survive by quickly detecting threat; (c) how feelings of fear and anxiety make us more likely to misperceive threat to be present when it is not; and (d) how when our perceptual systems are dealing with degraded or noisy data, they are more likely to make mistakes. Thus, a core aim of psychoeducation for hypervigilance AVH is to help a voice-hearer understand that our perceptions are influenced by what we expect to see and hear, and that when we expect to find threats in our environment, we are very likely to find them, even when they are not present.

The coping strategies suggested for hypervigilance AVH involve reducing physiological arousal, reducing perceived threat, reality testing, rational self-talk, and distraction. These coping strategies aim to help a person control feelings of fear and anxiety by either reducing bodily arousal (e.g., via progressive muscle relaxation) or their beliefs about the threats present in their environment (e.g., by discussing their beliefs with a trusted friend). If this is achieved, the likelihood that a service-user will experience a hypervigilance AVH should be reduced. Moreover, should they experience an AVH, their ability to control their levels of fear and anxiety should enable a service-user to engage in rational self-talk, where they can question whether what they have heard could really have been said to them, and/or to use distraction techniques to divert their attention away from scanning for threat and thus reduce AVH-related distress.

### Case vignette

Rick had been involved in a violent confrontation with a local gang, where he had tried to protect his father. He became very vigilant for any signs that he was to be targeted in a reprisal attack. He began to hear comments from people passing his house at night suggesting that he would be assaulted and this created a vicious circle where he stayed awake throughout the night to listen for signs of threat and began to hear more signs of this threat. This vicious circle was broken when Rick was hospitalized and began medication. On discharge he felt stigmatized by his mental health problems, remained convinced that he was in danger, and was, therefore, reluctant to leave his house. His voice hearing experiences were classified as hypervigilance AVH, as they occurred when his attention was externally-focused and their content was consistent with the threat he predicted he was under. Therapy focused on providing a longitudinal formulation of what had happened to Rick prior to his admission. The formulation highlighted that it would be natural for him to become more conscious of threat after the violent incident. Rick's situation was likened to a soldier in a dangerous situation where hypervigilance for threat has more positive than negative effects (i.e., the value of detecting genuine threats as early as possible outweighs the cost of making some false alarms). However, in Rick's situation, hypervigilance for threat had more negative than positive effects, and his sense of threat had escalated through sleep deprivation, substance misuse, and the onset of his voices. Psychoeducation included reviewing the importance of top-down processing or expectations on perception and error management theory. Rick found the formulation compelling and normalizing and it reduced the stigma he felt. Recognizing that the threatening comments he had heard were a result of him scanning his environment for threat, rather than genuine indicators of a threat, enabled him to reassess the level of danger he was in, allowing him to engage in graded exposure so that he was able to leave the house.

## Points of departure from traditional CBT for voice-hearing

The manual described here thus differs from traditional CBT for voice-hearing in that it provides multiple formulation templates that should aid the creation of a shared formulation concerning how a voice-hearer's AVH developed. These templates will reflect the individual factors for each voice-hearer (e.g., the specific role of abusive experiences, or of difficult family relationships, or of other stressful life events), but they guide the clinician to consider that varied cognitive/emotional processes may be driving different types of AVH. Thus, the clinician should be more able to (a) provide psycho-education that is a better “fit” with a voice-hearer's experiences, (b) identify behavioral experiments that are more likely help to change a voice-hearer's appraisals of their AVH, and (c) suggest coping strategies that are more likely to reduce the frequency of AVH. That being said, the approach we describe is not intended to “replace” existing CBT for voice-hearing; rather, its aim is to complement and enhance the options available to clinicians. We envisage it being used in tandem with other CBTp interventions with a specific focus, such as those that attempt to improve self-esteem (e.g., Freeman et al., 2014), or reduce compliance with commanding AVH (e.g., Birchwood et al., 2014).

## Problems with a subtyping approach

While there are reasons to believe that adopting the approach described here will lead to the development of more effective psychological interventions for voice-hearing, there are also a number of reasons to be cautious. First, there is a relatively long history of approaches that involve subtyping of hallucinatory experiences being of little practical use in terms of developing better interventions (Stephane, 2013). For example, Jaspers (1962) distinguished “true” AVH, which are heard in external space, from pseudohallucinations, which are heard in internal space (i.e., from inside the head), and suggested that the latter are a more benign form of AVH. However, it has been shown that this is not the case: internal and external AVH are equally distressing for voice-hearers (Copolov et al., 2004). Thus, one could argue that the present approach is yet another attempt to subtype AVH, which is unlikely to be of any practical value. While it is important to acknowledge this possibility, the present subtyping approach differs from some previous attempts to subtype AVH in that there is relatively strong theoretical (e.g., Ehlers and Clark, 2000; Fernyhough, 2004; Waters et al., 2006; Dodgson and Gordon, 2009) and empirical (e.g., Waters et al., 2006; Rapin et al., 2013; Dudley et al., 2014; Garwood et al., 2015) support for the three subtypes described here. This evidence indicates that the subtypes described here are related to separate cognitive processes, meaning that different interventions are likely to be required to help a voice-hearer cope with these different forms of AVH.

That being said, claims about these subtypes remain tentative and further research examining the subtypes of AVH described here is required. For example, it needs to be determined whether these subtypes can be reliably identified. While previous research (e.g., McCarthy-Jones et al., 2014b) employed existing measures to identify subtypes of AVH, it is likely that bespoke measures will need to be developed. In addition, research examining whether these subtypes of AVH are associated with different cognitive processes is required. For example, one would expect voice-hearers who experience inner speech-based AVH to report higher levels of dialogic inner speech as well as higher levels of inner speech that has the auditory qualities of another person's voice (as assessed by, e.g., the Varieties of Inner Speech Questionnaire, McCarthy-Jones and Fernyhough, 2011) than voice-hearers who do not experience inner speech-based AVH. In contrast, one would expect that voice-hearers who experience memory-based AVH to perform poorly on tasks involving the inhibition of unwanted memories (e.g., on Schnider and Ptak's, 1999, inhibition of currently irrelevant memories task) in comparison to voice-hearers who do not experience memory-based AVH. Finally, one would expect voice-hearers who experience hypervigilance AVH to show greater top-down influences on perception (e.g., using the jumbled speech task, Fernyhough et al., 2007, or the task employed in Daalman et al., 2012) than would voice-hearers who do not experience hypervigilance AVH. If these predictions hold true, it would provide support for the argument that different cognitive processes underlie different subtypes of AVH, which is consistent with the idea that different interventions may be required for the different subtypes. Clearly, however, the best way to investigate this claim would be to compare the efficacy of the manual described here with traditional CBT for AVH interventions (e.g., Morrison et al., 2004), as the most important step in establishing whether a subtyping approach is worthwhile would be to demonstrate that this approach is useful in clinical settings.

Another issue is that most voice-hearers report that they experience multiple subtypes of AVH. For example, McCarthy-Jones et al. (2014b) reported that the majority of their sample (59%) could be classified as experiencing more than one auditory hallucination subtype. One could claim, therefore, that it makes little sense to tailor CBT to the subtype of AVH a person reports when voice-hearers typically experience multiple subtypes. This claim can, however, be countered in a number of ways. First, it is important to emphasize that this approach aims to identify the subtype of AVH a person experiences, rather than aiming to subtype voice-hearers. In addition, it may be that, even in voice-hearers who report multiple subtypes of AVH, tailoring CBT to the subtypes they experience may be helpful. For example, it may prove helpful to work with a voice-hearer to establish that they experience two subtypes of AVH, to encourage them to employ different coping strategies when they experience different types of voices, and to ask them to focus on using the coping strategies to better control their most distressing voices first. Finally, it may be that experiencing only a single subtype of AVH is more common in people who have a short history of voice-hearing (e.g., who are experiencing their first episode of psychosis) and that, over time, multiple subtypes of AVH develop (see Jones, 2010, for a fuller account of this idea, which he calls the dynamic developmental progression of AVH). If this is the case, then a subtyping approach may be more appropriate for first episode or early intervention services.

A final concern is that the aims of this particular subtyping approach are to reduce the frequency of AVH and to reduce the distress associated with AVH. Aiming to reduce the frequency of AVH is, to some extent, inconsistent with one of the core tenet of CBT for psychosis: that clinicians should seek to reduce the distress associated with AVH by changing a voice-hearer's appraisals of their experience, and that reducing the frequency of psychotic experiences is not typically a target in therapy (Morrison and Barratt, 2010). Aiming to reduce the frequency of AVH is also at odds with the key values of the Hearing Voices Movement—a prominent, international user-led organization—who argue that interventions for AVH should encourage acceptance of voice-hearing, rather attempting to suppress it, or to reduce its frequency (Corstens et al., 2014). Despite this, many people with psychosis report that reducing the frequency of their AVH (or delusions) is a priority for them (e.g., Fischer et al., 2002; Rosenheck et al., 2005). This is true even for positive voices. For example, Jenner et al. (2008) reported that, in a sample of 138 participants who heard positive as well as negative voices, 57% did not want to keep their positive voices. The intervention we have described, therefore, may be suitable for voice-hearers who are seeking to reduce the frequency of their AVH, but may not suitable for voice-hearers who do not set this as a therapeutic goal.

## Conclusions

At present CBT for voice-hearing has only limited effectiveness. There is growing evidence that AVHs may be usefully divided into a set of subtypes and the existence of these subtypes might, in part, account for this limited effectiveness of CBT for voice-hearing. In this article we have described how CBT for voice-hearing could be tailored for three putative subtypes of AVH. At present, we are examining the acceptability of this approach for both clinicians and service-users and, if acceptability is demonstrated, we will investigate its efficacy in a randomized controlled trial.

### Conflict of interest statement

The authors declare that the research was conducted in the absence of any commercial or financial relationships that could be construed as a potential conflict of interest.
